# How marine cloud brightening could also affect stratospheric ozone

**DOI:** 10.1126/sciadv.adu4038

**Published:** 2025-05-14

**Authors:** Ewa M. Bednarz, James M. Haywood, Daniele Visioni, Amy H. Butler, Andy Jones

**Affiliations:** ^1^ Cooperative Institute for Research in Environmental Sciences (CIRES), University of Colorado Boulder, Boulder, CO, USA.; ^2^ NOAA Chemical Sciences Laboratory (NOAA CSL), Boulder, CO, USA.; ^3^ College of Engineering, Maths and Physical Science, University of Exeter, Exeter, UK.; ^4^ Met Office Hadley Centre, Exeter, UK.; ^5^ Department of Earth and Atmospheric Sciences, Cornell University, Ithaca, NY, USA.

## Abstract

Stratospheric ozone plays a crucial role in life and ecosystems on Earth, with a vast amount of research focused on the effects of human activities on ozone. Yet, impacts of tropospheric climate intervention methods like marine cloud brightening (MCB) have not previously been considered to reach the stratosphere. In this study, we demonstrate that MCB can also have a significant impact on both stratospheric and tropospheric ozone, and discuss the processes via which such an influence could occur. Our results demonstrate the inherent coupling between the troposphere and the stratosphere and underscore the need to assess not just the potential surface climate impacts of MCB, or any other climate intervention, but also their holistic interaction with the whole Earth system, including the middle atmosphere.

## Teaser

Impacts of tropospheric climate intervention methods can also reach the stratosphere and affect the ozone layer.

## INTRODUCTION

Stratospheric ozone plays a crucial role in life and ecosystems on Earth, shielding the surface from harmful ultraviolet B solar radiation. It is well understood that human activities can drive significant changes in this important atmospheric constituent. A full body of research exists that links past anthropogenic emissions of long-lived halogenated ozone-depleting substances, e.g., chlorofluorocarbons, with significant stratospheric ozone reductions over the second part of the 20th century. The discovery of the Antarctic ozone hole by Farman *et al.* in 1985 (*1*) in particular has sparked a considerable amount of scientific and political interest in the problem, and the resulting international implementation of the Montreal Protocol in 1987, and its subsequent adjustments and amendments, has successfully limited, or even completely phased out, the emissions of many potent ozone-destroying gases. As a result, stratospheric halogen levels are now decreasing and the ozone layer is recovering and is expected to return to its pre-1980 levels later this century (*2*). Aside from halogenated gases, stratospheric ozone concentrations are also affected by anthropogenic emissions of greenhouse gases, including carbon dioxide (CO_2_), methane (CH_4_), and nitrous oxide (N_2_O) (*3*–*5*), as well as a range of natural drivers including sulfate aerosols from explosive volcanic eruptions (*6*–*8*), stratospheric smoke from intense wildfires (*9*–*11*), and solar activity (*12*–*14*). Given its importance, continued monitoring of the stratospheric ozone layer, understanding drivers of any changes, and evaluating potential future risks under a range of anthropogenic emission scenarios continue to be the subject of quadrennial international assessments under the umbrella of the World Meteorological Organization.

Meanwhile, the emergence of adverse impacts of climate change combined with a growing probability of a future overshoot of surface temperature levels deemed “safe” by other international accords like the Paris Agreement has fueled research into alternative temporary measures to offset some of the most negative impacts of climate change, so-called “climate intervention” methods. To date, the most prominent proposals in the scientific literature are stratospheric aerosol injection (SAI) and marine cloud brightening (MCB). Both methods rely on increasing Earth’s reflectivity of incoming solar radiation to cool surface temperatures. SAI involves injecting sulfate aerosols (or their gaseous precursors) into the lower stratosphere, resulting in a cooling mechanism analogous to that observed after large explosive volcanic eruptions (*15*–*17*). MCB, on the other hand, is applied in the troposphere and involves introducing additional particles into the low-level cloud decks over the ocean to increase cloud reflectivity (*18*–*20*).

Given that SAI involves introducing new particles into the stratosphere and thus directly affects its composition and climate, and given evidence from the impacts from volcanic eruptions, it is widely recognized that potential SAI deployment could alter the ozone layer and influence its projected long-term recovery (*21*). In common with other aerosols, sulfate provides active surfaces on which heterogeneous halogen and nitrogen reactions can rapidly occur that would otherwise occur only very slowly in the gas phase; these change atmospheric concentrations of species that can react with and catalytically deplete ozone (*22*). In addition, aside from cooling the troposphere, SAI also warms the lower stratosphere as sulfate aerosols absorb near-infrared radiation; this effect can drive changes in stratospheric circulation and transport, thereby also affecting ozone through changes in atmospheric dynamics (*23*).

In contrast to SAI, MCB is applied far away from the stratosphere and has not been previously considered as something that can affect stratospheric ozone. Here, we use the UK Earth System Model (UKESM; see Materials and Methods) to demonstrate that MCB could have a significant impact on both stratospheric and tropospheric ozone and discuss the processes by which the influence would occur. We use the MCB simulation (“G6mcb”) introduced by Haywood *et al.* (*24*) that injects sea salt aerosols into four Pacific Ocean regions most susceptible to MCB to bring down global mean surface temperatures from the Shared Socioeconomic Pathway (SSP) (*25*) SSP5-8.5 levels to the levels simulated in the analogous middle-of-the-road SSP2-4.5 simulation. The MCB scenario has been designed to be directly comparable with two other existing simulation protocols constituting part of the Geoengineering Model Intercomparison Project (GeoMIP) (*26*): the G6sulfur experiment that assesses the impacts from SAI and the G6solar experiment that uses an idealized reduction in solar constant (“solar dimming”), which could also be viewed as a space-based climate intervention method. The use of such a setup allows us to demonstrate that ozone changes simulated under this particular MCB realization are distinct and do not merely mirror those occurring under the other two most commonly studied climate intervention methods.

This particular MCB scenario uses a high-end global warming baseline (as do G6sulfur and G6solar). In addition, studies have shown that regional MCB could affect the El Niño–Southern Oscillation (ENSO) and its teleconnections (*27*–*29*). Thus, the strategy for deployment that we investigate here, where susceptible clouds are targeted only on the eastern side of the Pacific Ocean, could be viewed as a nonjudicious MCB deployment owing to its significant ENSO response (*24*). As such, we do not aim to project future ozone changes under a more moderate MCB scenario or under a more plausible deployment strategy but rather aim to illustrate and highlight the processes by which such an MCB influence on ozone could occur.

## RESULTS

### Simulated ozone changes under MCB

**Fig. 1.**
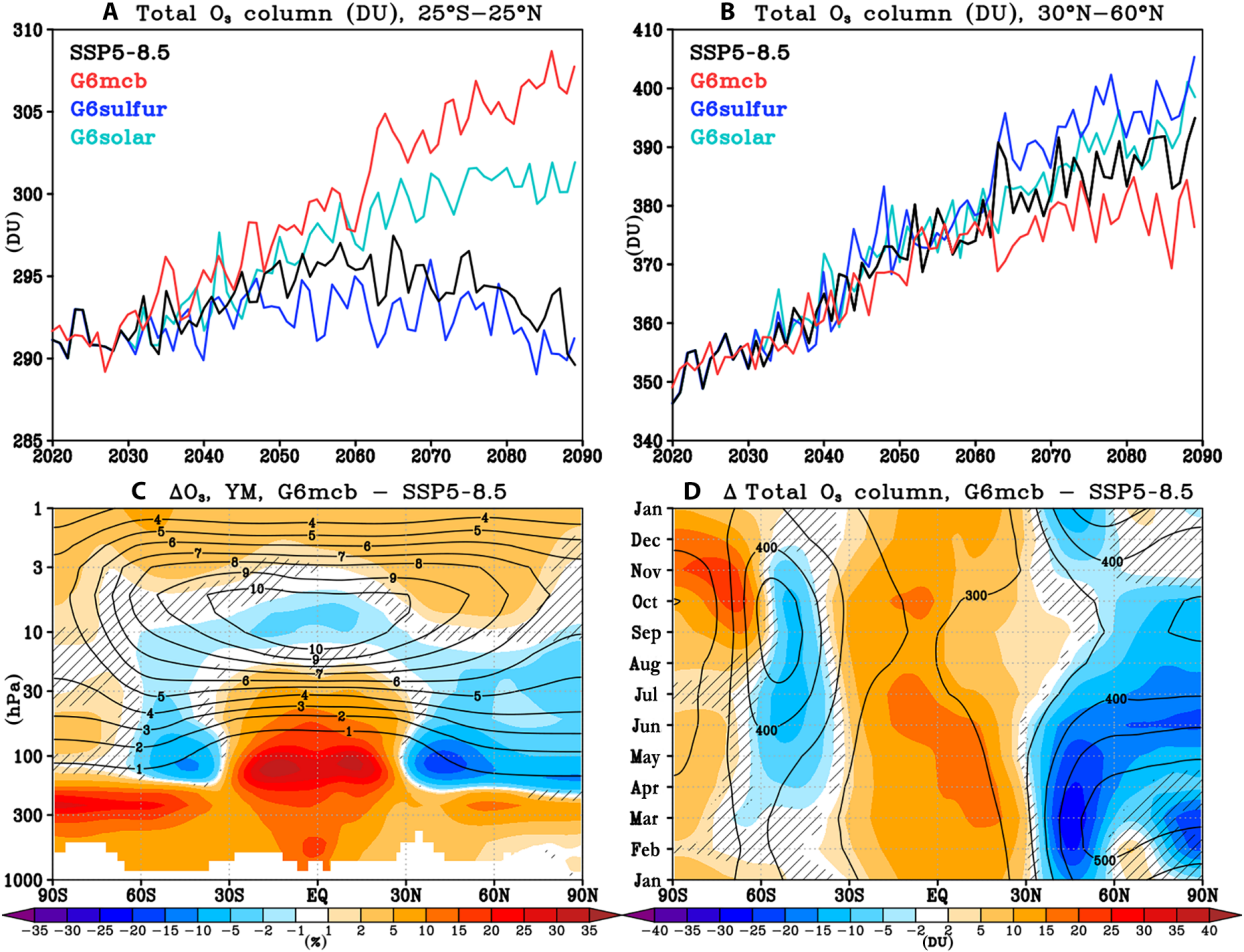
Simulated ozone changes under different climate intervention scenarios. Top: time series of yearly mean ensemble mean (**A**) tropical and (**B**) NH midlatitude total ozone columns in the greenhouse gas–only SSP5-8.5 simulation and the three climate intervention scenarios (G6mcb, G6sulfur, and G6solar). Bottom: changes in the late 21st century (2070 to 2089) (**C**) yearly mean zonal mean ozone mixing ratios (%) and (**D**) monthly mean total ozone column (DU) between G6mcb and SSP5-8.5 (shading). Contours show the corresponding values in SSP5-8.5 (2070 to 2089) for reference. Hatching marks areas where the response is not statistically significant (defined here as smaller than ± 2 standard errors in the difference in means). See figs. S1 and S2 for the corresponding changes in G6solar and G6sulfur.

Cooling of the upper stratosphere by increased greenhouse gases (GHGs) and the reduction in stratospheric halogen levels by the implementation of the Montreal Protocol and its later amendments and adjustments is projected to increase ozone over the 21st century (*2*). In accord, the simulated tropical total ozone columns increase over the first half of the 21st century under all scenarios considered here (Fig. 1A). Under the high-end climate change scenario (SSP5-8.5) with no climate intervention, the tropical total ozone column is projected to then decrease in the second half of the 21st century as the impact of the GHG-induced acceleration of tropical upwelling and, thus, enhanced input of ozone-poor tropospheric air into the lower stratosphere dominates over the impact of decreasing halogen levels and the GHG-induced upper stratospheric cooling (*4*, *5*). Under the MCB scenario (G6mcb), the total ozone column continues to increase in the tropics throughout the 21st century, with ~15 DU more ozone than under climate change alone simulated by the year 2090. This increase in tropical total ozone column under MCB is not seen in the SAI scenario (which instead shows a slight reduction in tropical ozone) and is also significantly larger than simulated under the same level of global mean surface cooling achieved via idealized solar reduction (G6solar). This tropical ozone column increase under MCB corresponds to a year round ozone increase throughout the tropical lower stratosphere and in the troposphere (Fig. 1, C and D). The free tropospheric ozone increase in particular, while constituting a relatively smaller contribution to the total ozone column, is not found under either the SAI or solar dimming scenario (fig. S1) but has important implications for air quality as well as acts as positive radiative forcing (because in the troposphere, ozone acts as a greenhouse gas), thereby partially offsetting the direct cooling effects from MCB.

Unlike in the tropics, the Northern Hemisphere (NH) midlatitude total ozone column continues to increase throughout the 21st century under climate change alone (SSP5-8.5, Fig. 1B). This is because in the extratropics, the GHG-induced acceleration in the large-scale Brewer-Dobson circulation (BDC) acts to increase local ozone levels as the result of enhanced transport of ozone from its tropical production region to the mid- and high latitudes. Under MCB, on the other hand, the NH midlatitude total ozone column is significantly reduced compared to ozone under climate change alone (by ~10 DU in yearly mean by the year 2090) and begins to stabilize over the later part of the 21st century. This behavior contrasts with the relative increase in the NH midlatitude total ozone column projected under SAI (compared to SSP5-8.5) or the very small ozone change simulated under solar dimming. This NH midlatitude total ozone column decrease under MCB maximizes in boreal winter and spring (Fig. 1D; see also fig. S3) and is accompanied by a similar, albeit smaller, total ozone column decrease in the Southern Hemisphere (SH) midlatitudes in austral winter and spring. Last, at higher southern latitudes, MCB leads to a significant springtime polar ozone increase of up to ~20 DU (Fig. 1C; see also fig. S3). In the following sections, we discuss in detail the different dynamical and chemical processes that contribute to these distinct ozone changes under MCB. While the focus here is on MCB changes in particular, we present the corresponding changes diagnosed for the SAI and solar dimming in the Supplementary Materials for comparison.

### Dynamical drivers of ozone changes under MCB

**Fig. 2.**
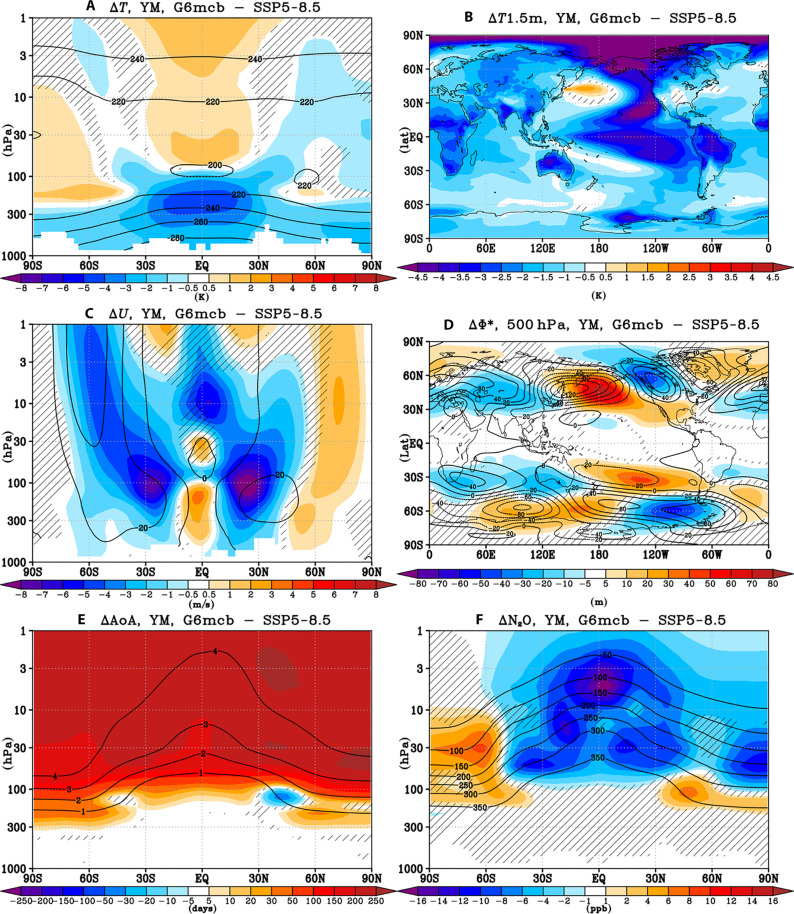
Dynamic drivers of the simulated ozone response to MCB. Shading: yearly mean late 21st century (2070 to 2089) changes in (**A**) zonal mean temperature (K), (**B**) near-surface air temperature (K), (**C**) zonal mean zonal wind (m/s), (**D**) eddy geopotential height at 500 hPa, (**E**) zonal mean age of air (days), and (**F**) zonal mean N_2_O mixing ratios [parts per billion (ppb)] between G6mcb and SSP5-8.5. Contours show the corresponding values in SSP5-8.5 for reference [for (E), these are in the units of years]. Hatching as in Fig. 1. See figs. S4 to S9 for the corresponding changes in G6solar and G6sulfur.

Changes in tropospheric temperatures can affect atmospheric planetary wave generation and propagation into and within the stratosphere, affecting stratospheric and tropospheric circulation and transport, with distinct effects attributed to uniform and regionally inhomogeneous surface temperature changes (*30*, *31*). By design, all climate intervention methods cool the troposphere (Fig. 2A and fig. S4) and surface (Fig. 2B and fig. S5) compared to the climate change–only scenario. Because of the feedback with water vapor changes (see the Chemical drivers of ozone changes under MCB section), in the free troposphere, the cooling tends to maximize in the tropical upper troposphere, weakening the subtropical jets in both hemispheres (Fig. 2C and fig. S6). Changes in the position of the critical line in the lower stratosphere as the result of subtropical jet weakening affect planetary wave propagation, reducing wave breaking in the subtropical and extratropical stratosphere (fig. S10) and hence decelerating the large-scale BDC (fig. S10) [see also (*32*)]. This manifests as a substantial increase in mean age of air throughout most of the stratosphere (Fig. 2E). The reduction in upwelling in the tropical upper troposphere and lower stratosphere contributes to the increase in tropical stratospheric ozone simulated under MCB, as less ozone-poor tropospheric air is transported into the stratosphere. In the extratropics, this is also associated with reduced transport of ozone from its photochemical production region in the tropical midstratosphere to higher latitudes, thus acting to decrease ozone levels in the extratropics.

Regional MCB strategies in particular have been linked with changes in equatorial Pacific sea surface temperatures and ENSO variability (*28*, *29*, *33*), and this particular MCB realization used here gives rise to a strong La Niña–like response in the eastern Pacific (Fig. 2B) [see also (*24*)]. Modeling and reanalysis studies have linked variability in ENSO to changes in tropical atmospheric upwelling, stratospheric residual circulation, mixing, and ozone (*34*–*37*). In accord, changes in equatorial Pacific near-surface temperatures simulated in the MCB simulations drive a La Niña–like Rossby wave train into the extratropics that in the NH deconstructively interferes with the climatological quasistationary wave pattern, driving a significant reduction in vertical wave propagation from the troposphere to the stratosphere (Fig. 2D) [e.g., (*38*, *39*)]. The decreased flux of wave activity entering the stratosphere under MCB further decelerates the stratospheric BDC and alters ozone transport. In addition, the La Niña–like near-surface temperature changes further weaken the subtropical jets (*40*–*42*). As subtropical jets generally act as a mixing barrier, their weakening drives an enhancement in horizontal mixing in the extratropical lower stratosphere in the MCB simulations. This is diagnosed by the localized decreases in age of air (Fig. 2E), as more young tropospheric air enters the extratropical lower stratosphere, and by the localized increases in N_2_O (Fig. 2F), as more N_2_O-rich tropospheric air enters lower stratospheric extratropics. These anomalies become particularly strong in certain seasons, e.g., from boreal spring to summer (figs. S3, S11, and S12). The enhanced lower stratospheric mixing under MCB further contributes to the ozone increases in the tropical lower stratosphere and the ozone decreases in the midlatitudes (Fig. 1, C and D) from the weakened BDC.

**Fig. 3.**
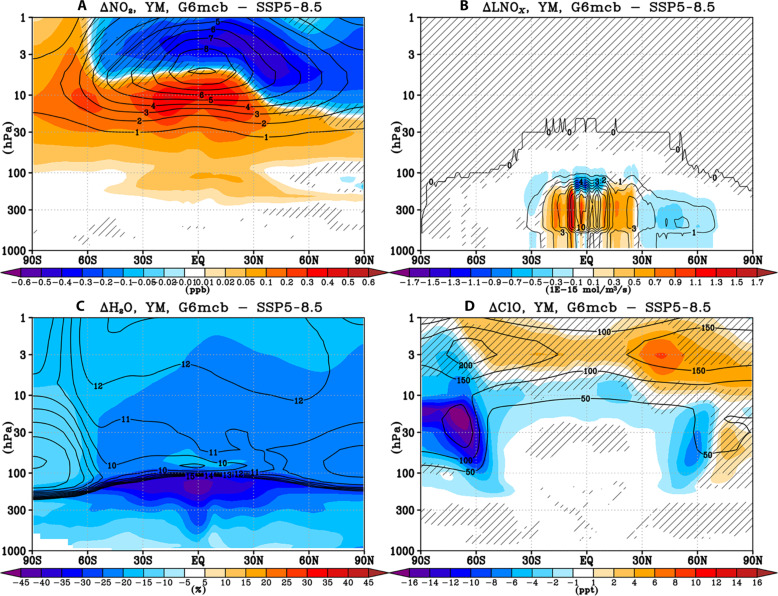
Chemical drivers of the simulated ozone response to MCB. Shading: yearly mean late 21st century (2070 to 2089) changes in zonal mean (**A**) NO_2_ mixing ratios (ppb), (**B**) lightning NO*_x_* production rate (10^−15^ mol/m^3^/s), (**C**) water vapor (%), and (**D**) ClO [parts per trillion (ppt)] between G6mcb and SSP5-8.5. Contours show the corresponding values in SSP5-8.5 for reference [in units of ppm for (C)]. Hatching as in Fig. 1. See figs. S14 to S17 for the corresponding changes in G6solar and G6sulfur.

Last, the reduction in tropospheric wave activity flux under MCB also strengthens the NH stratospheric polar vortex in winter and spring (Fig. 2C and fig. S13). This acts to reduce ozone in the NH polar regions from winter to spring (Fig. 1D and fig. S3) because of the reduction in mixing with midlatitude air as well as enhanced heterogeneous processing under colder polar temperatures (fig. S19). A similar effect is not found in the SH, where the stratospheric polar vortex decelerates instead (particularly in austral spring; fig. S13) as the result of changes in meridional temperature gradients under upper tropospheric cooling, leading to an increase in springtime Antarctic ozone of up to ~20 DU (Fig. 1D) because of both enhanced in-mixing and less heterogeneous halogen processing (Fig. 3D and fig. S19). Similar Antarctic vortex and ozone changes are found also under uniform solar dimming (figs. S2A and S6A).

### Chemical drivers of ozone changes under MCB

Apart from driving changes in ozone transport, changes in atmospheric temperatures and circulation under MCB can also affect atmospheric concentrations of chemical species of importance to ozone chemistry. First, the MCB simulation shows a significant increase in active nitrogen concentrations (NO*_x_*; here represented as NO_2_ in Fig. 3A) in the tropical upper troposphere and lower stratosphere (below ~50 hPa). This NO*_x_* increase likely arises from the enhanced tropospheric NO*_x_* production from lightning (Fig. 3B and fig. S18D) associated with the MCB-induced increase in convective precipitation over land (fig. S18A). The MCB-induced increase in NO*_x_* in the tropical upper troposphere and lower stratosphere acts to increase ozone levels in this region in a manner analogous to that occurring in the polluted troposphere, thus contributing to the significant ozone increases simulated in the tropics under MCB (Fig. 1, A, C, and D). Increased lower stratospheric ozone production under higher NO*_x_* levels has also been found in the context of increased tropospheric N_2_O emissions (*43*–*46*). Above, in the middle stratosphere (~50 to 5 hPa), MCB drives a tropical NO*_x_* increase as the result of the large-scale weakening in transport by the BDC (see the Dynamical drivers of ozone changes under MCB section; accompanied by NO*_x_* decreases in the upper stratosphere), and this effect chemically reduces ozone locally in the middle stratosphere (~10 hPa; Fig. 1C), i.e., where the NO*_x_*-catalyzed gas phase ozone loss dominates ozone chemistry.

Second, tropospheric cooling under MCB drives a substantial reduction in water vapor (Fig. 3C), both in the troposphere (as colder tropospheric air holds less moisture) and in the stratosphere (as the tropical tropopause cools and facilitates more dehydration). This reduction in water vapor acts to increase ozone concentrations both in the troposphere (*47*) and in the upper stratosphere (*48*) because of reduction in O(^1^D) quenching and HO*_x_*-mediated gas phase ozone loss. Last, unlike the MCB-induced changes in nitrogen species and water vapor, MCB impacts on ozone caused by any changes in stratospheric halogens and their activation are likely relatively small because of much lower halogen levels in the late 21st century. Nonetheless, the simulated changes in the strength of the stratospheric polar vortices (Fig. 2C and fig. S13) are associated with consistent changes in heterogeneous halogen activation in spring (so enhanced halogen activation inside the stronger NH polar vortex and less halogen activation inside the weaker SH polar vortex; Fig. 3D for annual mean changes and fig. S19 for seasonal changes). This effect, while small, still contributes to the reduced Arctic and enhanced Antarctic ozone levels simulated in each respective spring under MCB.

## DISCUSSION

Anthropogenic influences on past, present, and future evolution of stratospheric ozone have important consequences for ultraviolet-related human and ecosystem health impacts. While anthropogenic emissions of halogenated ozone-depleting substances, greenhouse gases, and as of lately, climate intervention using SAI continue to be the focus of in-depth scientific research, tropospheric MCB—since proposed to be applied far away from the stratosphere—has not previously been considered to cause changes reaching the stratosphere. In this study, we have demonstrated that MCB can have a significant impact on both stratospheric and tropospheric ozone. This influence manifests itself via a combination of changes in atmospheric circulation and transport as the result of both large-scale tropospheric cooling and regional patterns of anomalous sea surface temperatures, in particular those associated with the modulation of the ENSO variability. In addition, coupling of the MCB-induced changes in atmospheric temperatures and circulation to concentrations of atmospheric species directly important for ozone chemical production and loss rates plays a further role, including the enhanced ozone production in the tropical upper troposphere and lower stratosphere as the result of concurrent increases in lightning NO*_x_* under MCB-induced changes in tropospheric convective precipitation. While some of those processes would also operate under other climate intervention methods (for instance, those driven by the large-scale reduction of global mean surface temperatures), the MCB simulations project net ozone changes under MCB that are distinct from those projected under SAI or solar dimming, underscoring the fine interplay of different ozone drivers and their changes and the need to assess them in general for all proposed climate intervention methods. We note that as with the simulations of SAI or climate change, the exact responses are subject to many uncertainties and are likely to be model dependent. Moreover, additional processes not currently included in the Earth System Model used here could also be important under MCB, for instance, the enhanced heterogeneous processing on sea salt used for MCB and its impact on tropospheric chemistry and radiative forcing (*49*), emphasizing the need for future research in this area.

While the particular MCB realization used in this study can be considered nonjudicious and quite extreme because of its significant impacts on ENSO variability and its resulting teleconnections [see (*24*) for a more in-depth discussion], we used it here as it was designed to be comparable to the SAI scenario that has been analyzed in depth for the WMO 2022 Ozone Assessment (*21*). Furthermore, because other studies have also noted a strong La Niña response to different MCB deployments, we expect that the mechanisms we highlighted would apply somewhat independently of the specific scenario and strategy used, as long as MCB is used to offset a substantial portion of global warming. Future research should try to understand which parts of the response are strategy dependent and could thus be minimized through the design of an optimized MCB seeding strategy, similar to what has been done for SAI in Bednarz *et al.* (*50*). Such an approach has been suggested in Chen *et al.* (*27*), although the efficacy of the MCB cooling effect is reduced if cloud regimes other than the highly susceptible stratocumulus deck in the eastern ocean basins are targeted. Ultimately, our study proves that—just as for SAI—analyses of the potential risks and benefits of MCB (or all other emerging climate intervention methods) should not just focus on changes in surface climate but should also include assessments of changes in the middle atmosphere and the ozone layer, demonstrating the unavoidable interlink between different atmospheric layers and the need for providing a holistic picture of any climate intervention method.

## MATERIALS AND METHODS

### UK Earth System Model (UKESM1)

UKESM1 (*51*) is a state-of-the-art earth system model developed jointly by the UK Met Office and a number of British universities. It consists of the UK Unified Model in its Global Atmosphere 7.1 configuration (*52*), including a horizontal resolution of 1.25 latitude by 1.875 longitude, 85 vertical levels, and a model top at ~85 km and featuring comprehensive tropospheric-stratospheric chemistry (*53*), modal GLOMAP aerosol module (*54*), coupled ocean model (*55*), interactive sea ice (*56*), ocean biogeochemistry (*57*), and land model (*58*). Its simulations participated in numerous international modeling activities, including phase 6 of the Coupled Model Intercomparison Project (CMIP6) and phase 2 of the Chemistry Climate Model Initiative (CCMI-2022).

### GeoMIP G6solar and G6sulfur simulations

The protocol for the GeoMIP G6 simulations is described in (*26*). The simulations span 2015 to 2100, with the CMIP6 SSP5-8.5 scenario (*25*) as the underlying GHG emission scenario. Both G6 simulations, consisting of three ensemble members each, reduce global mean surface temperatures from the SSP5-8.5 levels to the levels simulated in the corresponding middle-of-the road SSP2-4.5 scenario using either uniform SO_2_ injection between 10°S-10°N at 18 km (“G6sulfur”) or an idealized reduction in the solar constant (“G6solar”). The results from those simulations participated in numerous studies, including those informing the WMO ozone depletion 2022 report (*21*, *59*).

### UKESM G6mcb simulation

The UKESM G6mcb simulation, described in more detail in Haywood *et al.* (*24*), has been designed to be directly comparable to the G6solar and G6sulfur simulations. In particular, it also uses the SSP5-8.5 GHG scenario and aims to reduce the global mean surface temperatures to the SSP2-4.5 levels, but it does so by injecting sea salt aerosols into the eastern seaboard of the Pacific Ocean. The choice of strategy was to target susceptible marine clouds that occur to the west of continents owing to the Ekman-pumping forced low sea surface temperatures, which are conducive to the formation of stratocumulus clouds. However, previous studies (*60*, *61*) with forerunners of the UKESM1 model have shown that targeting the South East Atlantic can lead to detrimental impacts on precipitation over the Amazon rainforest, results that have been confirmed in multimodel simulations (*19*). Thus, only the eastern seaboard of the Pacific Ocean was targeted. The size distribution of the emitted sea salt aerosol was optimized to provide the largest cooling efficiency. A dry radius of 86 nm was found to be optimal for the sea salt concentrations needed to provide the significant global mean radiative forcing of around −4 W m^−2^ by the end of the century (i.e., the difference in the radiative forcing between SSP2-4.5 and SSP5-8.5), although dry radii as small as 36 nm were found to be effective if offsetting weaker radiative forcings in common with process modeling studies (*62*, *63*). Emissions of sea salt were made into the lowest model layer centered at 20-m altitude and were assumed to be constant across the grid boxes in the designated areas. Full details are provided in (*24*). As in G6solar and G6sulfur, the experiment consists of three ensemble members.
