# Supplementary material for: How marine cloud brightening could also affect stratospheric ozone
Source: Sci Adv. 2025 May 14;11(20):eadu4038. doi: 10.1126/sciadv.adu4038 (PMC12077500; doi:10.1126/sciadv.adu4038)
Supplement: Supplementary file 1 — Figs. S1 to S19 [file sciadv.adu4038_sm.pdf]

Science Advances
AAAS

Supplementary Materials for

How marine cloud brightening could also affect stratospheric ozone

Ewa M. Bednarz *et al.*

Corresponding author: Ewa M. Bednarz, ewa.bednarz@noaa.gov

Sci. Adv. **11**, eadu4038 (2025)
DOI: 10.1126/sciadv.adu4038

This PDF file includes:

Figs. S1 to S19

Supplementary Figures

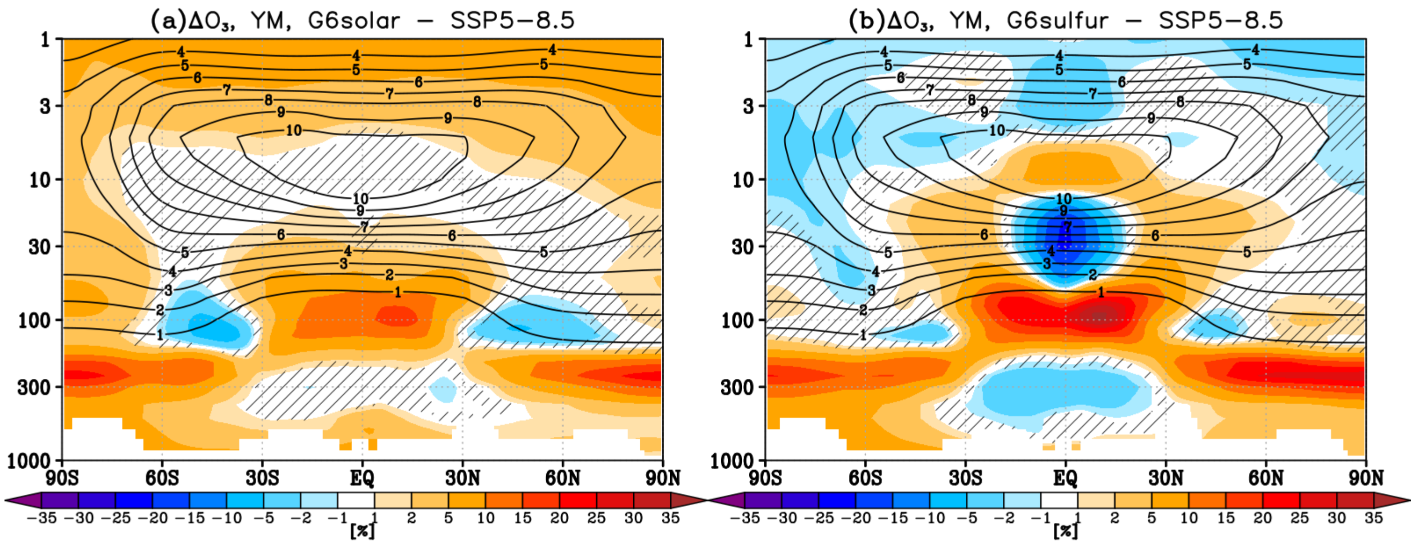

Figure S1. As in Figure 1c of the main manuscript but for changes in (a) G6solar and (b) G6sulfur.

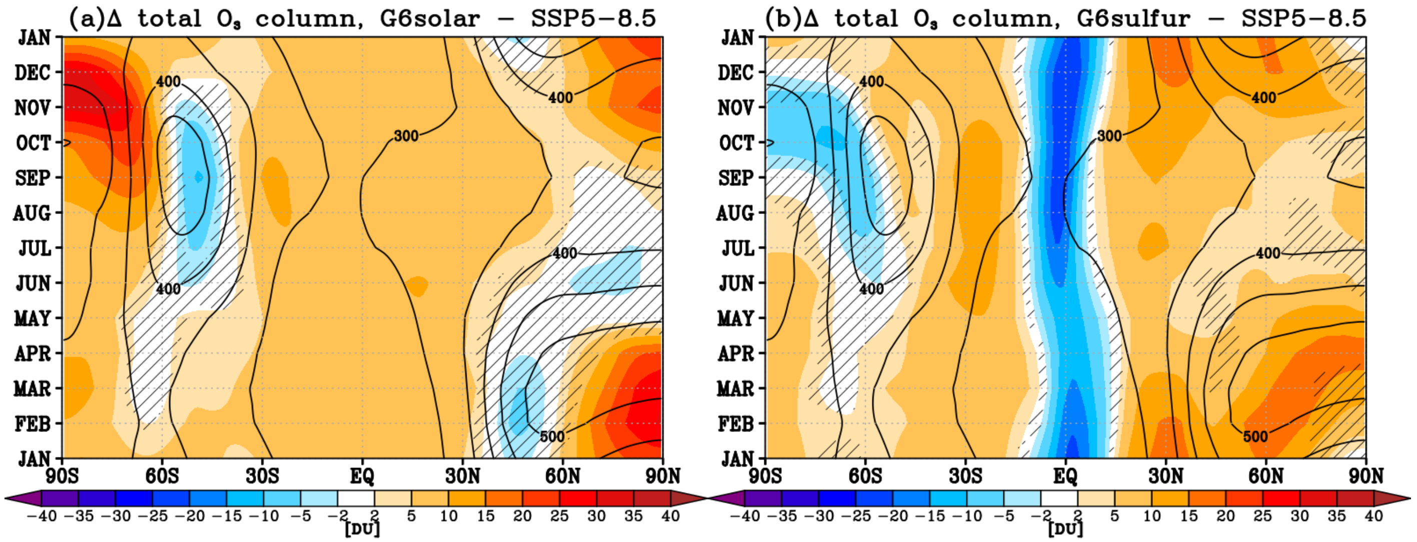

Figure S2. As in Figure 1d of the main manuscript but for changes in (a) G6solar and (b) G6sulfur.

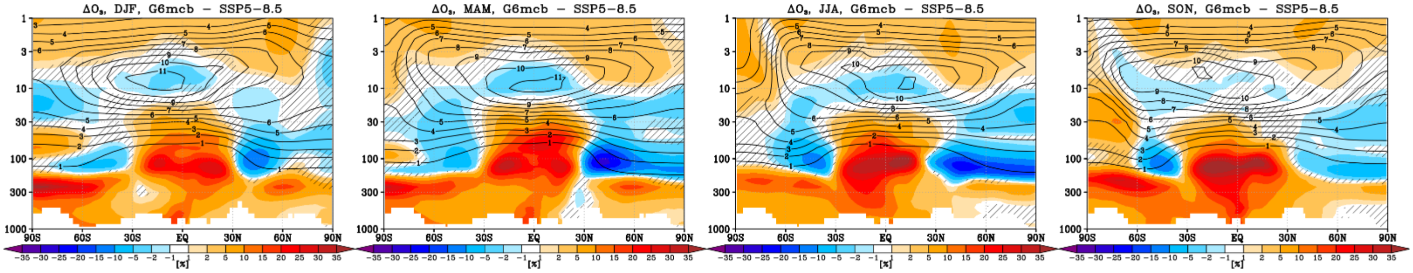

Figure S3. As in Figure 1c of the main manuscript but for seasonal mean (DJF, MAM, JJA and SON) changes.

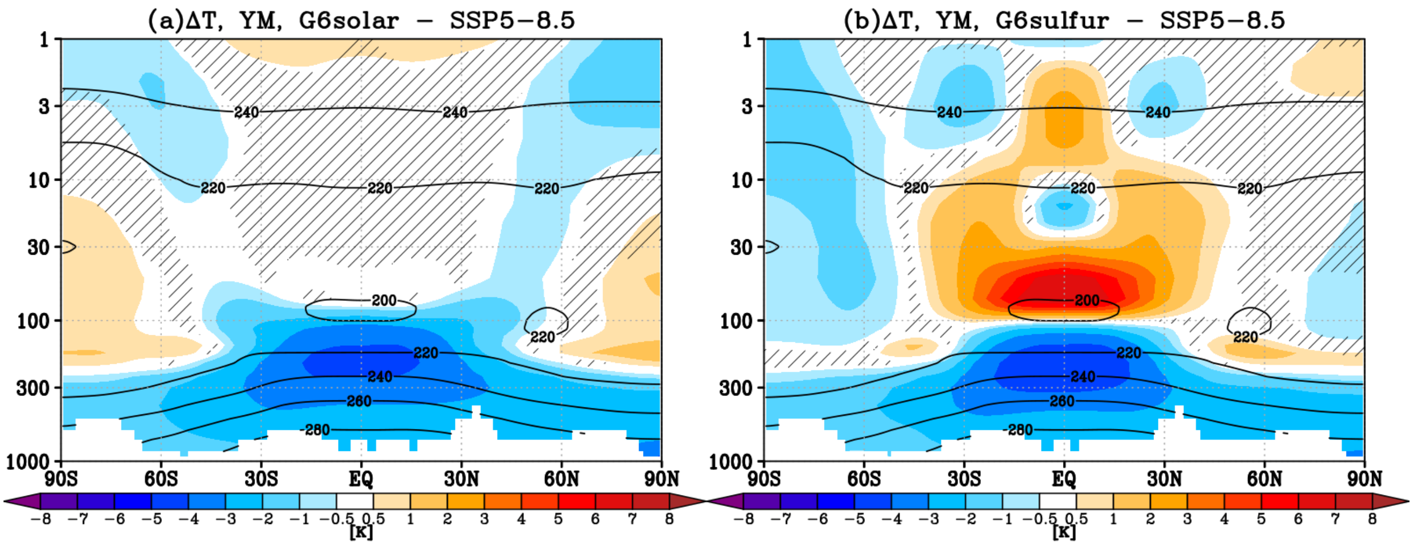

Figure S4. As in Figure 2a of the main manuscript but for changes in (a) G6solar and (b) G6sulfur.

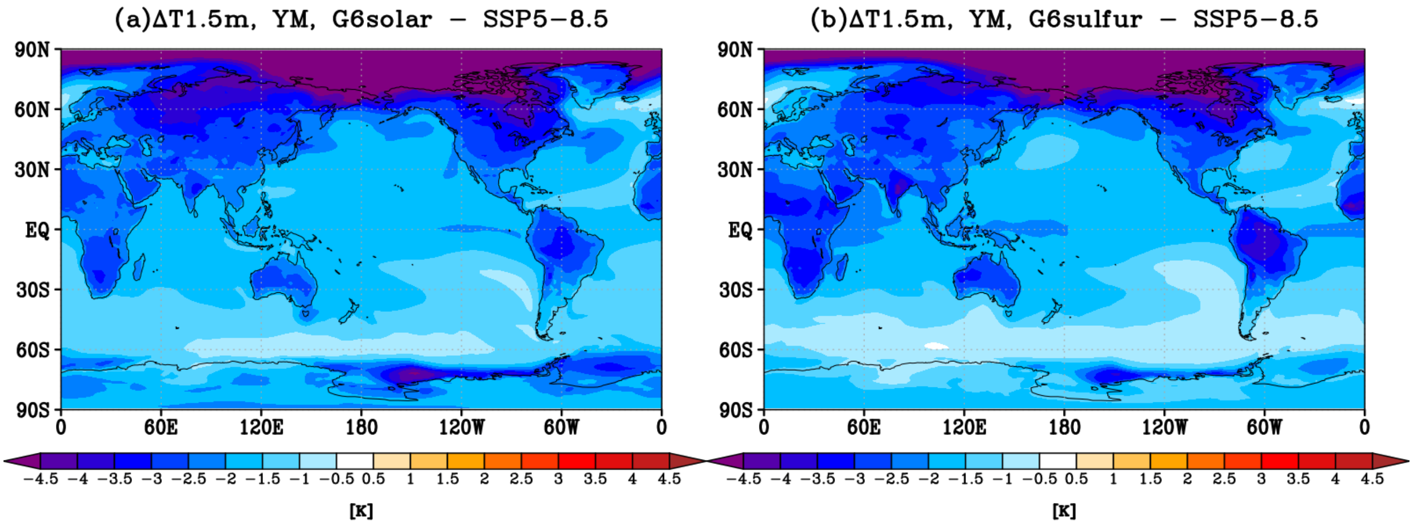

Figure S5. As in Figure 2b of the main manuscript but for changes in (a) G6solar and (b) G6sulfur.

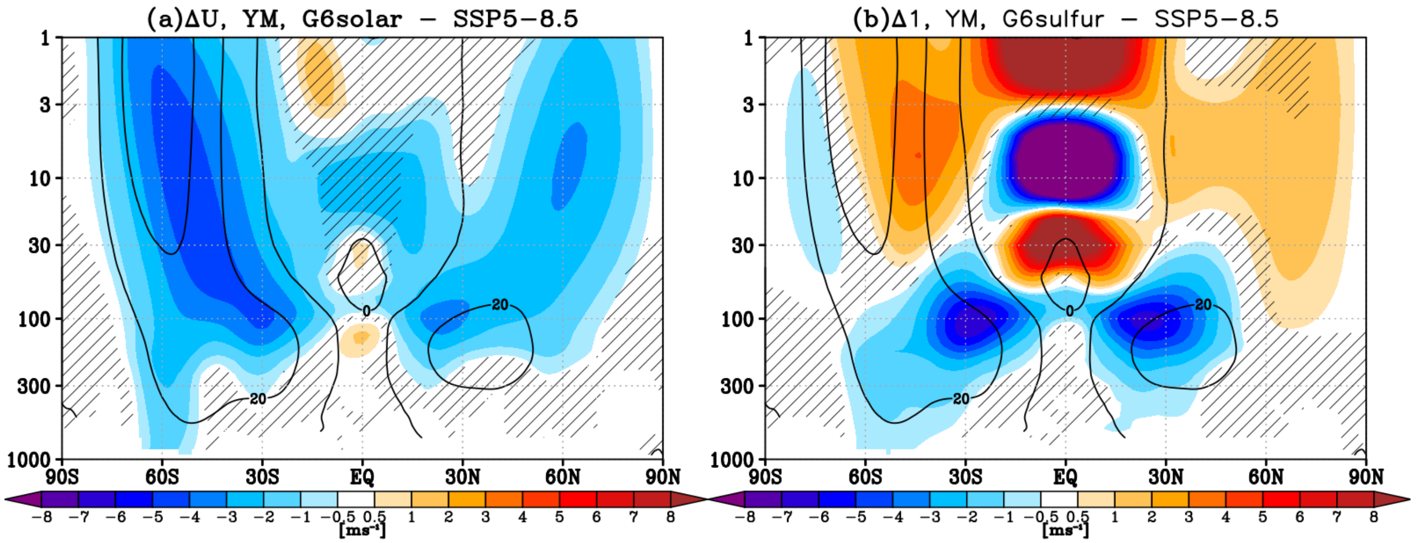

Figure S6. As in Figure 2c of the main manuscript but for changes in (a) G6solar and (b) G6sulfur.

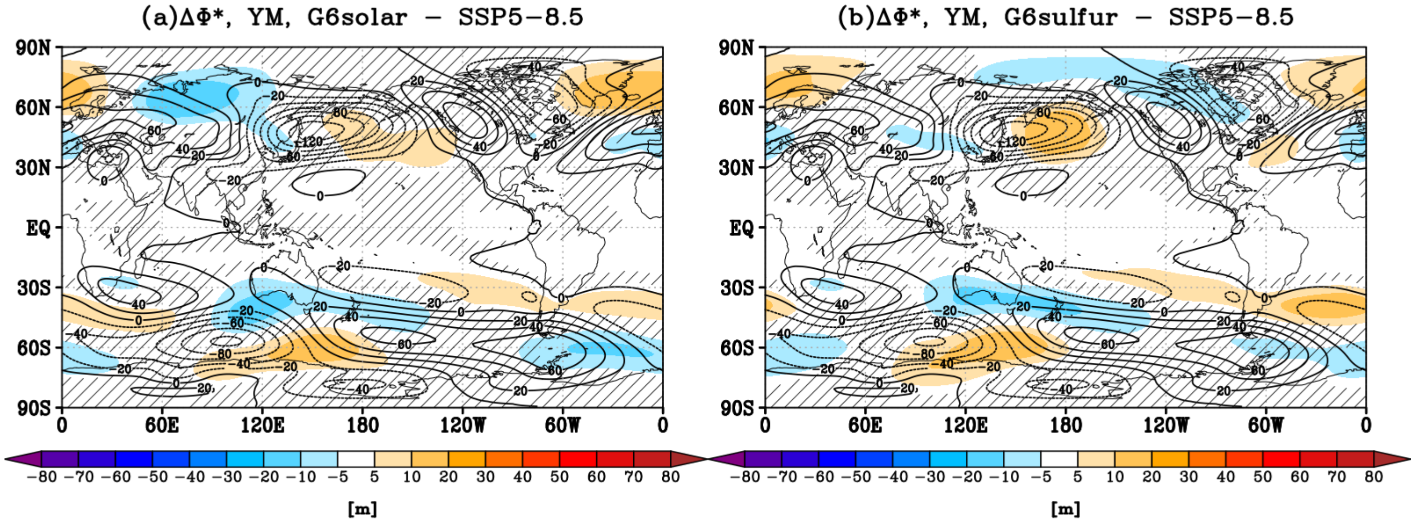

Figure S7. As in Figure 2d of the main manuscript but for changes in (a) G6solar and (b) G6sulfur.

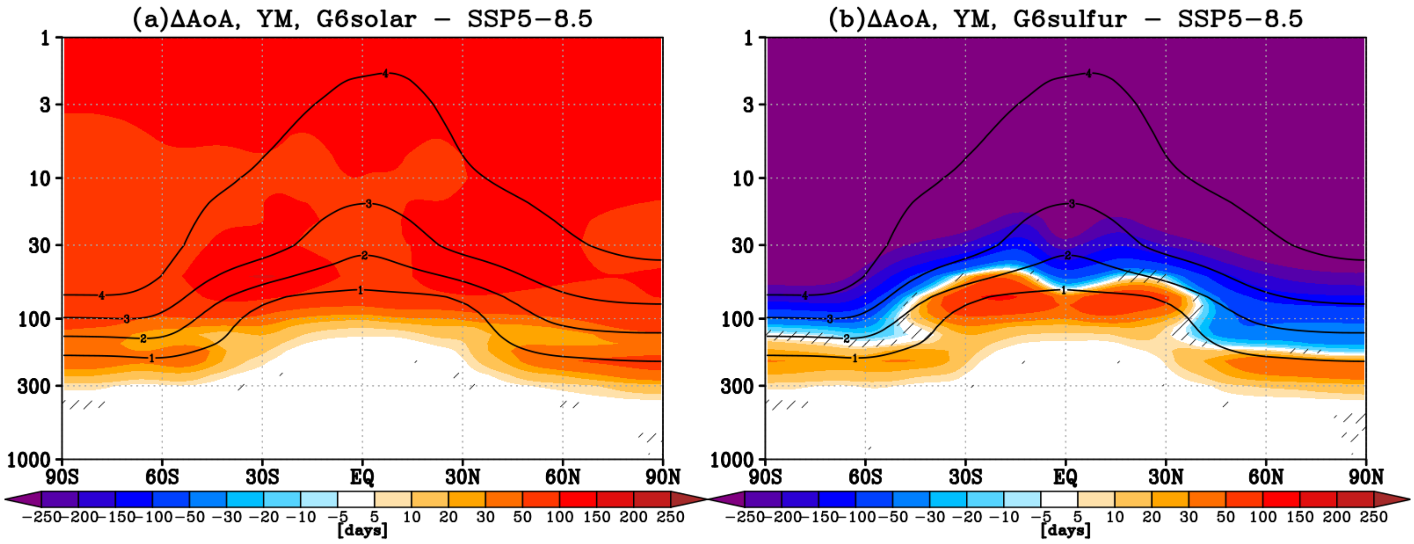

Figure S8. As in Figure 2e of the main manuscript but for changes in (a) G6solar and (b) G6sulfur.

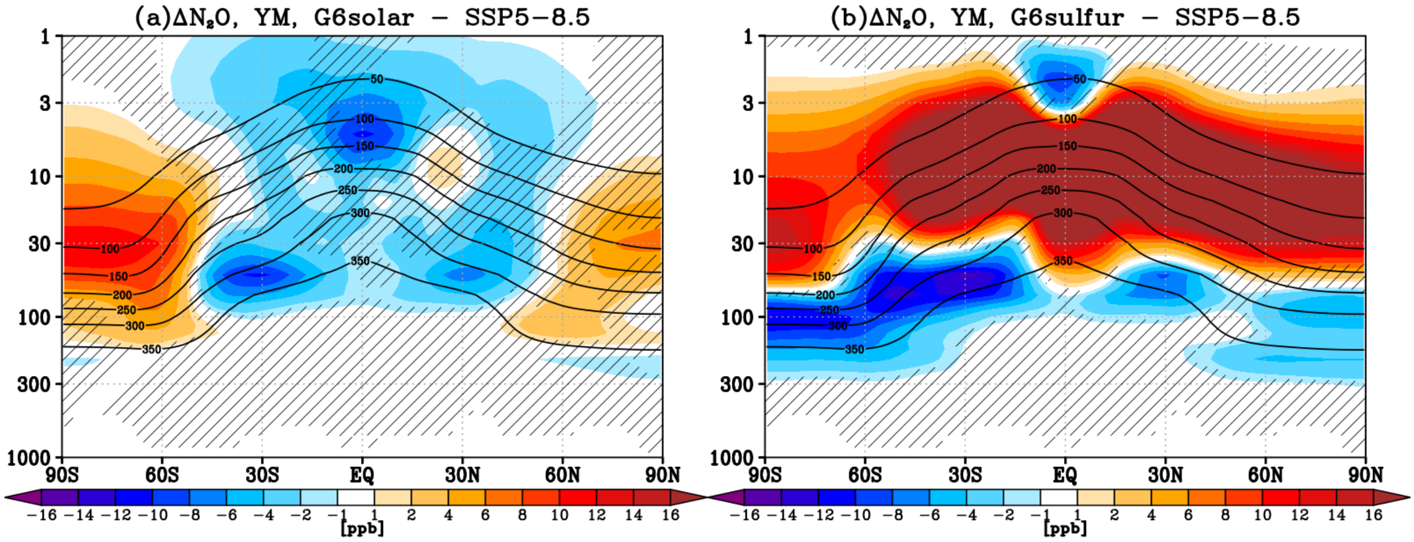

Figure S9. As in Figure 2f of the main manuscript but for changes in (a) G6solar and (b) G6sulfur.

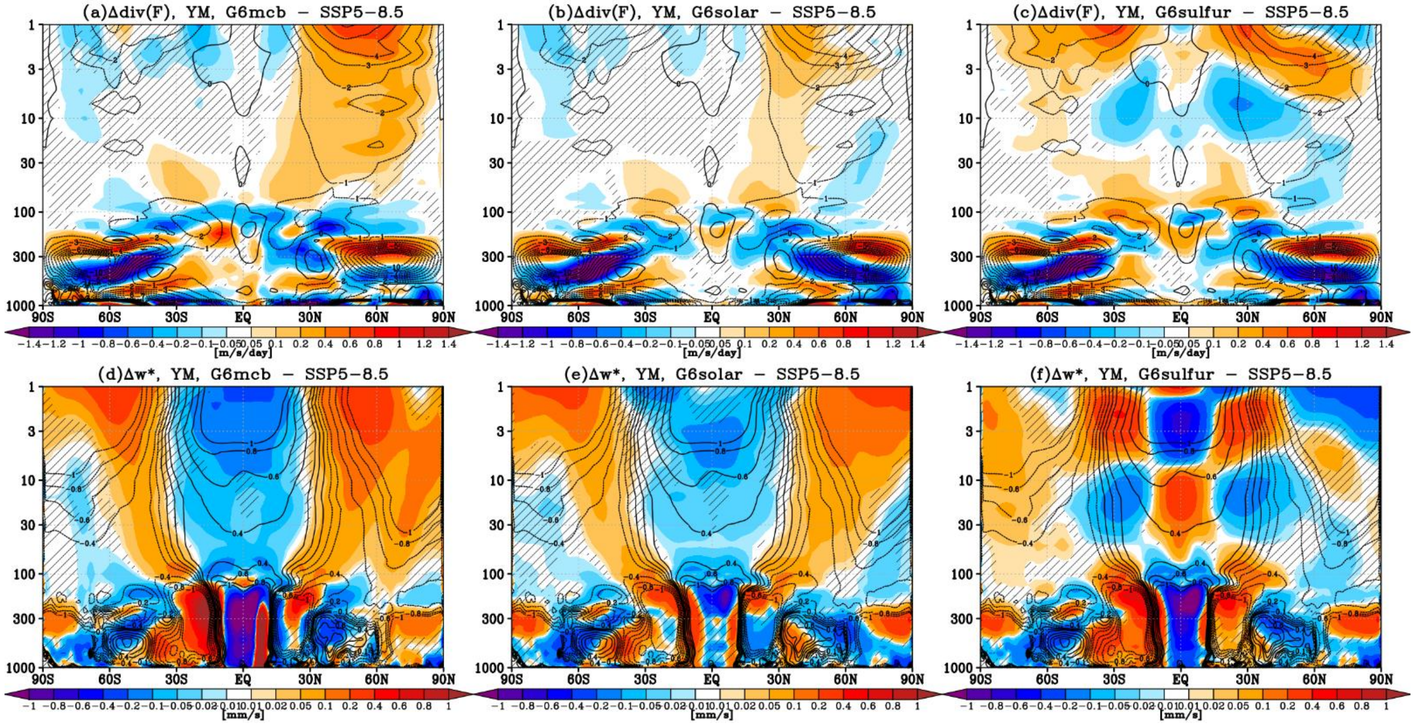

Figure S10. Shading: Yearly mean late 21st century (2070-2089) changes in (a-c) EP flux divergence and (d-f) vertical component of the TEM residual circulation between the different climate intervention scenarios (G6mcb, G6solar, G6sulfur) and SSP5-8.5. Contours show the corresponding values in SSP5-8.5 for reference. Hatching marks areas where the response is not statistically significant (defined here as smaller than +/- 2 standard errors in the difference in means).

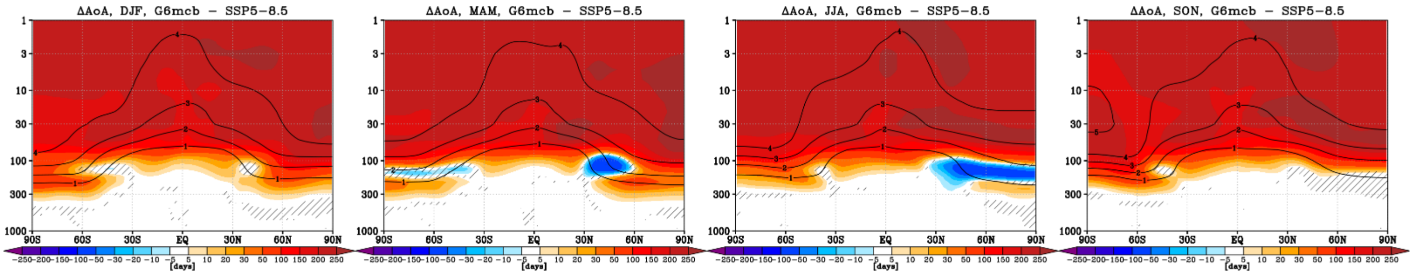

Figure S11. As in Figure 2e of the main manuscript but for seasonal mean (DJF, MAM, JJA and SON) changes.

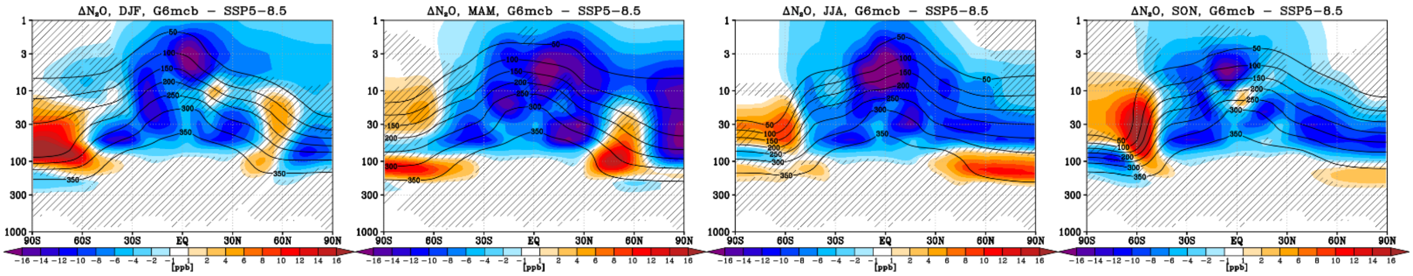

Figure S12. As in Figure 2d of the main manuscript but for seasonal mean (DJF, MAM, JJA and SON) changes.

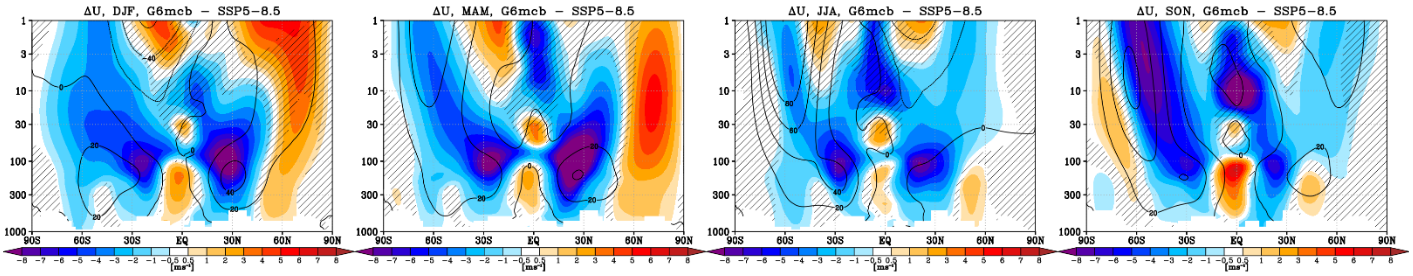

Figure S13. As in Figure 2c of the main manuscript but for seasonal mean (DJF, MAM, JJA and SON) changes.

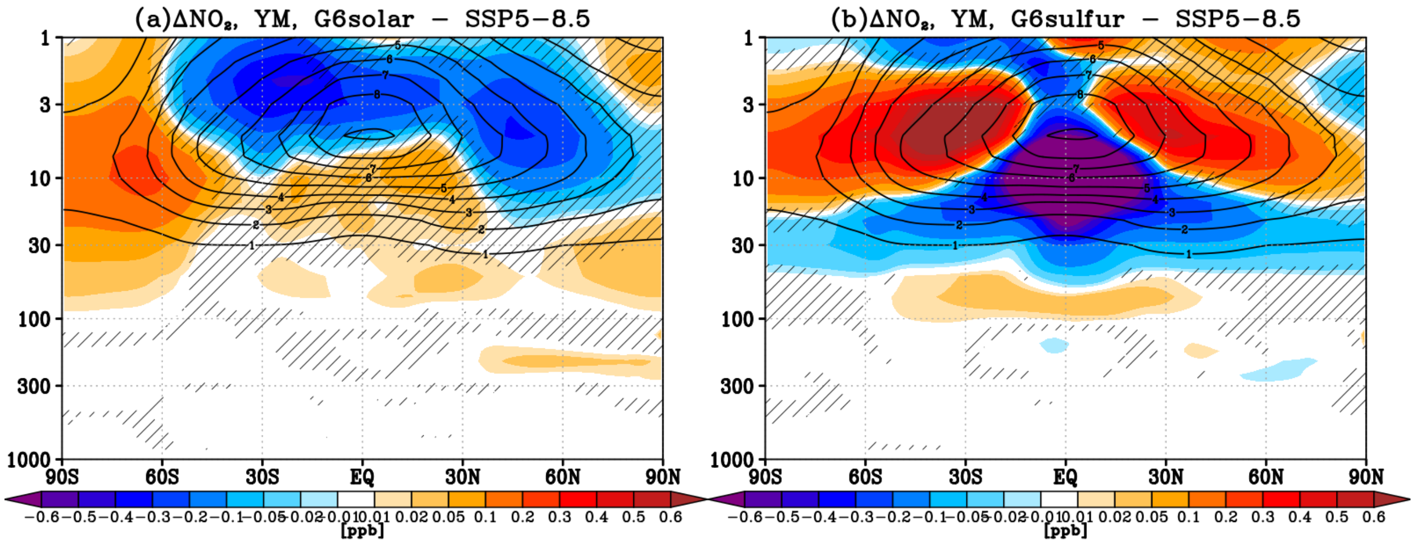

Figure S14. As in Figure 3a of the main manuscript but for changes in (a) G6solar and (b) G6sulfur.

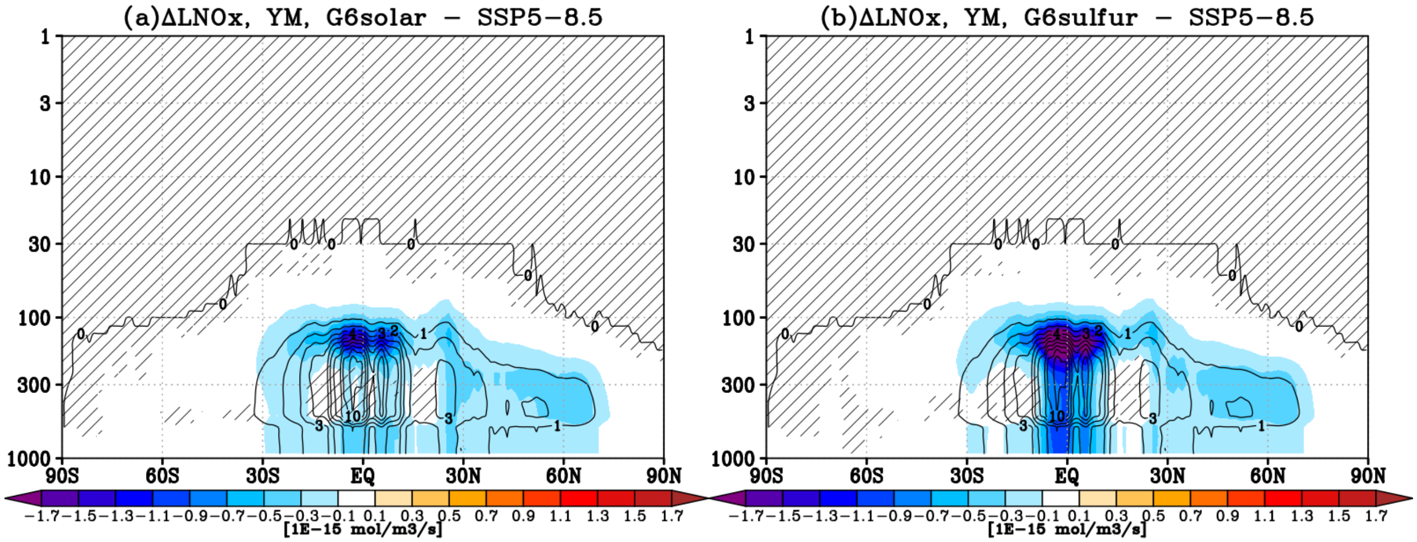

Figure S15. As in Figure 3b of the main manuscript but for changes in (a) G6solar and (b) G6sulfur.

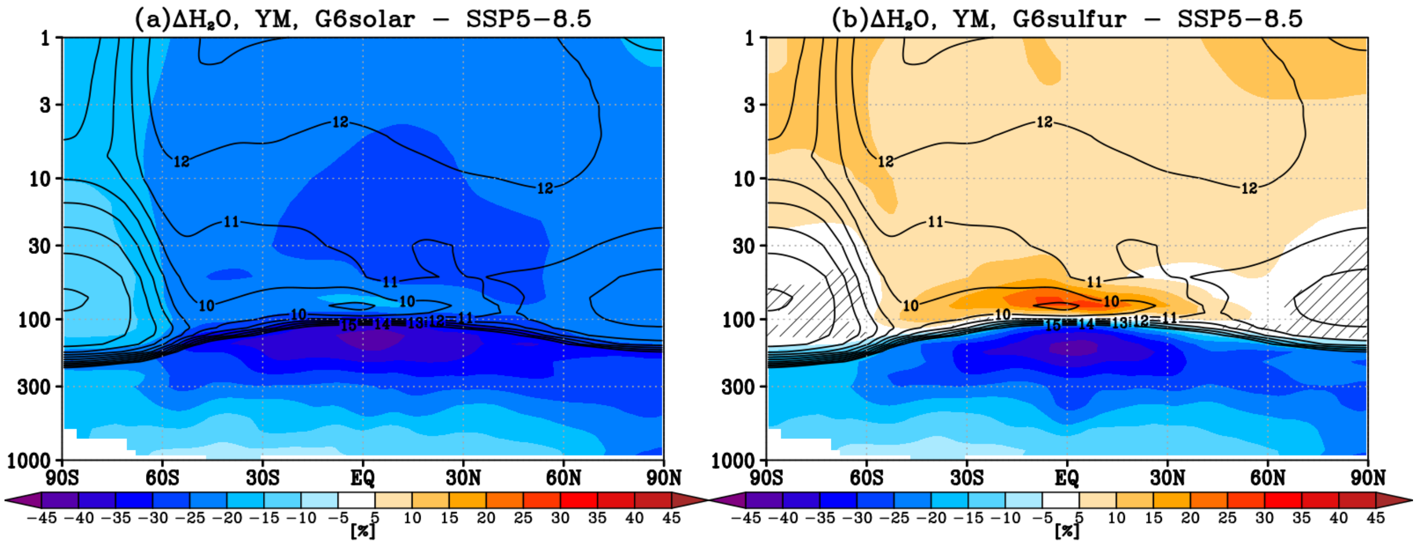

Figure S16. As in Figure 3c of the main manuscript but for changes in (a) G6solar and (b) G6sulfur.

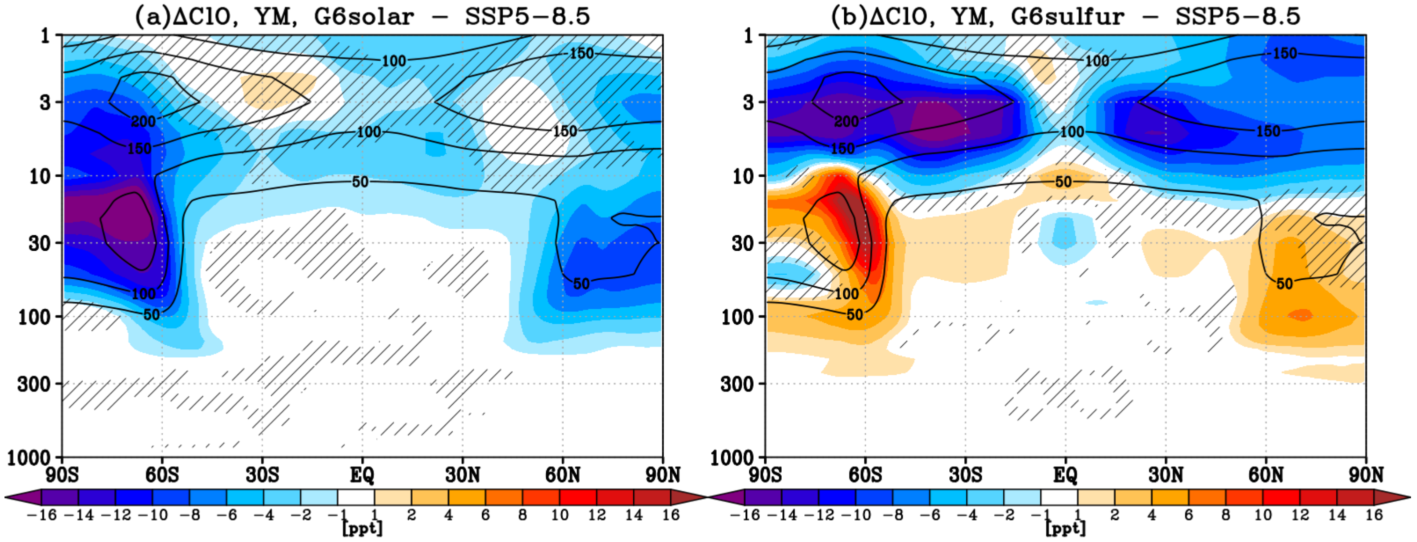

Figure S17. As in Figure 3d of the main manuscript but for changes in (a) G6solar and (b) G6sulfur.

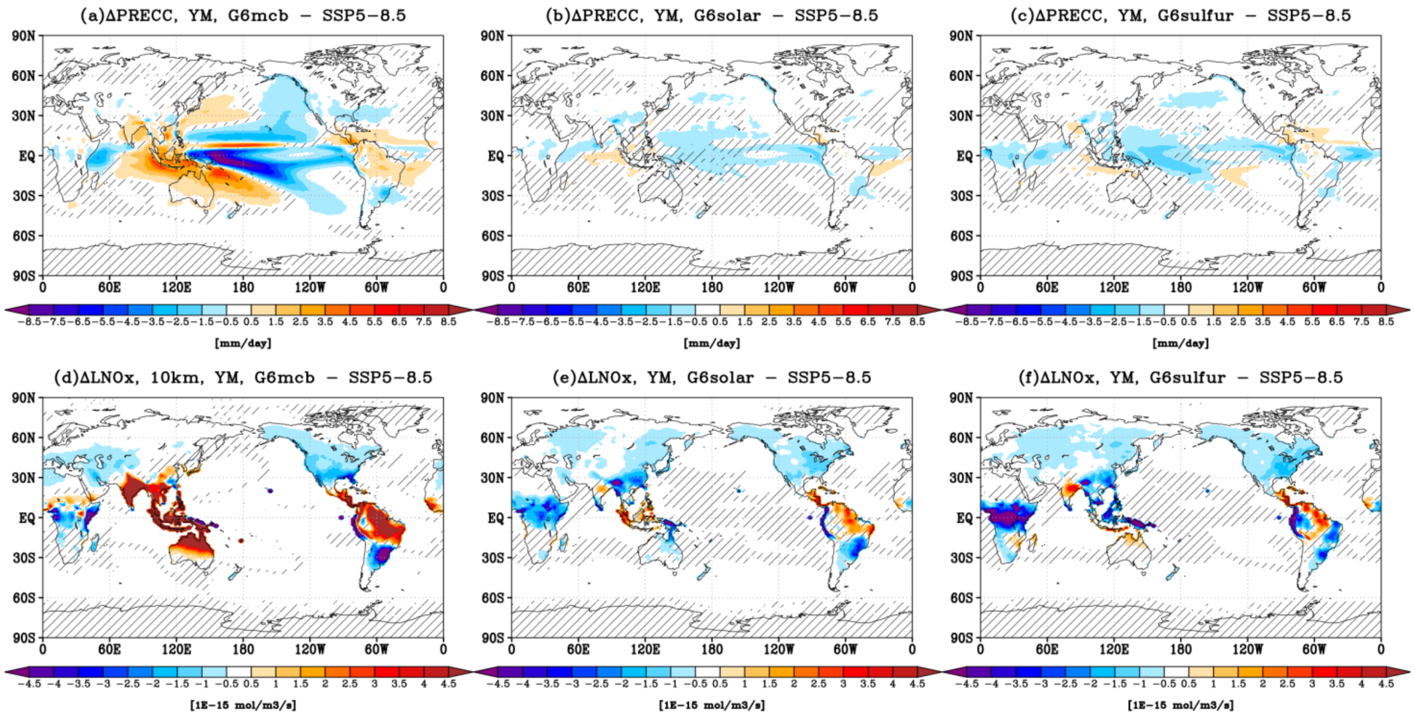

Figure S18. Shading: Yearly mean late 21st century (2070-2089) changes in (a-c) convective precipitation and (d-f) lightning NOx production at 10 km between the different climate intervention scenarios (G6mcb, G6solar, G6sulfur) and SSP5-8.5. Contours show the corresponding values in SSP5-8.5 for reference. Hatching marks areas where the response is not statistically significant (defined here as smaller than +/- 2 standard errors in the difference in means).

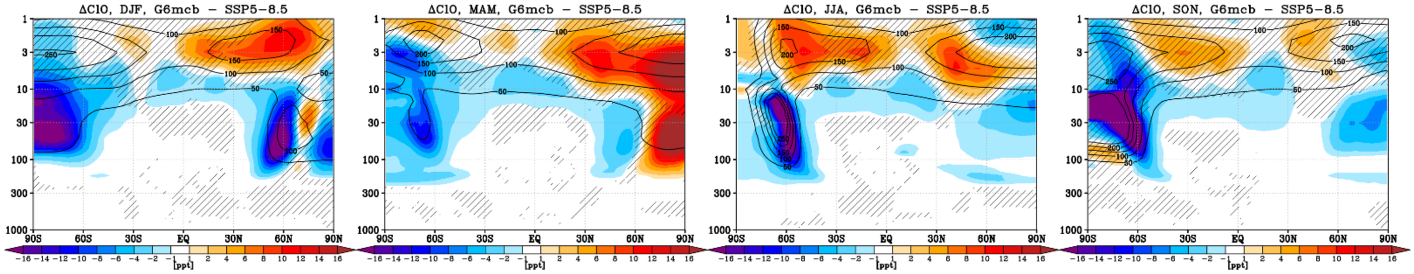

Figure S19. As in Figure 3d of the main manuscript but for seasonal mean (DJF, MAM, JJA and SON) changes.
